# Systematic review and validation of clinical models predicting survival after oesophagectomy for adenocarcinoma

**DOI:** 10.1093/bjs/znac044

**Published:** 2022-03-02

**Authors:** Piers R Boshier, Alison Swaray, Bhamini Vadhwana, Arun O’Sullivan, Donald E Low, George B Hanna, Christopher J Peters

**Affiliations:** 1 Department of Surgery and Cancer, Imperial College London, London, UK; 2 Department of Thoracic Surgery, Virginia Mason Medical Centre, Seattle, Washington, USA

## Abstract

**Background:**

Oesophageal adenocarcinoma poses a significant global health burden, yet the staging used to predict survival has limited ability to stratify patients by outcome. This study aimed to identify published clinical models that predict survival in oesophageal adenocarcinoma and to evaluate them using an independent international multicentre dataset.

**Methods:**

A systematic literature search (title and abstract) using the Ovid Embase and MEDLINE databases (from 1947 to 11 July 2020) was performed. Inclusion criteria were studies that developed or validated a clinical prognostication model to predict either overall or disease-specific survival in patients with oesophageal adenocarcinoma undergoing surgical treatment with curative intent. Published models were validated using an independent dataset of 2450 patients who underwent oesophagectomy for oesophageal adenocarcinoma with curative intent.

**Results:**

Seventeen articles were eligible for inclusion in the study. Eleven models were suitable for testing in the independent validation dataset and nine of these were able to stratify patients successfully into groups with significantly different survival outcomes. Area under the receiver operating characteristic curves for individual survival prediction models ranged from 0.658 to 0.705, suggesting poor-to-fair accuracy.

**Conclusion:**

This study highlights the need to concentrate on robust methodologies and improved, independent, validation, to increase the likelihood of clinical adoption of survival predictions models.

## Teaser

This study examines different models predicting long-term survival of patients with oesophageal cancer and assesses their performance in real-world data.

## Introduction

Globally, oesophageal cancer affects 746 000 patients and is associated with 459 000 deaths annually^1,2^. While squamous cell carcinoma (SCC) remains the predominant global histological subtype, in recent years, the incidence of oesophageal adenocarcinoma has exceeded that of SCC in many parts of North America and Europe^3^.

Surgical resection of the oesophagus, with or without chemoradiotherapy or chemotherapy, offers the principal curative treatment modality for oesophageal adenocarcinoma but is only appropriate in a minority of patients who present with localized disease. For those patients who do undergo potentially curative surgical resection, overall 5-year survival is typically between 20 and 30 per cent and seldomly exceeds 50 per cent^4,5^. The associated morbidity and long-term sequelae of oesophageal resection serve as additional obstacles in the treatment of oesophageal cancer. The desire to establish greater equipoise of risk and benefit in the surgical management of oesophageal adenocarcinoma has meant that the ability to accurately predict survival after oesophagectomy is of particular clinical significance.

The eighth edition of The American Joint Committee on Cancer (AJCC) and the International Union for Cancer Control (UICC) TNM classification, is principally based on anatomical tumour extent and remains the most widely adopted method of prognostication in oesophageal cancer^6^. Nevertheless, TNM staging criteria does not acknowledge other pathological, demographic, and clinical variables that are also known to impact upon survival^7,8^. In oesophageal cancer, as in other malignancies, predictive models of survival have been developed in an effort to improve prognostication. Such models are intended to support clinical decision-making and to better inform patients of their envisaged disease outcome. However, it is notable that few of these models are ever routinely used in clinical practice.

This systematic review aimed to identify published clinical and pathological models that predict survival in patients undergoing potentially curative surgical resection for oesophageal adenocarcinoma. Where possible, the performance of identified models were assessed using a prospectively collected multicentre dataset.

## Methods

### Search strategy

This systematic review was conducted in accordance with the recommendations of the Cochrane Library and MOOSE guidelines^9^. A systematic literature search using the Ovid Embase and MEDLINE databases (from 1947 to 11 July 2020) was performed to identify studies reporting predictive models of survival in oesophageal adenocarcinoma. Details of the search strategy are provided in *Table S1*. The titles and abstracts of identified articles were screened by three independent reviewers (A.S., P.R.B., and B.V.) for potentially relevant studies that were subsequently subject to full-text review. To identify further potentially relevant studies, the reference lists of included articles were hand searched.

Inclusion criteria were studies that developed or validated a clinical prognostication model to predict either overall or disease-specific survival in patients with oesophageal adenocarcinoma undergoing surgical treatment with curative intent. Models that included patients with both adenocarcinoma and SCC were included if the former was the predominant tumour subtype used to develop the model. Likewise, studies including patients receiving therapies with curative intent, other than surgery, were included, if surgery was the predominant treatment modality within the study cohort. No restrictions were made regarding patient demographics, surgical approach, use of (neo)adjuvant therapies, or study design. Models developed for either pre- or postoperative use were also included. Models that included experimental metabolic and/or genetic biomarkers that are either not currently routinely available or used within clinical practice were excluded. Models that were developed through the use of artificial neural networks were also excluded, owing to their unsuitability for independent validation. Non-English-language articles and conference abstracts without an associated published full-text article were excluded. Any disagreement regarding a study’s inclusion was resolved by a fourth reviewer (C.P.). Three reviewers (A.S., B.V., and A.O’S.) independently extracted data from included studies.

### Definitions

Oesophageal adenocarcinoma was defined as a histologically specific malignancy affecting the oesophagus and/or gastroesophageal junction (Siewert type I and II) and oesophagectomy as surgery to resect all or part of the oesophagus through either an open, hybrid, or totally minimally invasive approach but not including endoscopic techniques. A prognostic model was defined as a multivariable tool designed to predict patient survival (overall or disease specific) or a surrogate factor that was shown by the authors to correlate directly and reliably with patient survival.

### Methodological quality assessment

The methodological quality of the included studies was assessed using the Critical Appraisal and Data Extraction for Systematic Reviews of Prediction Modelling Studies (CHARMS) checklist^10^. This checklist groups 35 key items into 11 domains that may be extracted from individual studies for the purpose of critical appraisal.

### Model validation

Models were validated using an independent dataset of 2450 patients who underwent oesophagectomy for oesophageal adenocarcinoma with curative intent. Data were acquired from the Oesophageal Cancer Clinical and Molecular Stratification Consortium (OCCAMS; 1088 patients)^11,12^, Predicting Outcomes of Esophageal Malignancy Biomarker Consortium (POEM; 811 patients), and from a high-volume North American Centre (Virginia Mason Medical Center; 551 patients). Local institutional review board approval was obtained by all participating centres for the purpose of sharing anonymised data. Characteristics of the validation dataset are provided in *Table 1*.

**Table 1:** Characteristics of validation dataset

	Validation cohort (*n* = 2450)		Available data/2450	
**Mean (s.d.) age (years)**	64.5 (10.5)		2442 (99·7)	
**Sex (M:F)**	2078:372 (84.8:15.2)		2450 (100.0)	
**Mean (s.d.) BMI**	27.5 (4.9)		841 (34.3)	
**Median (i.q.r.) CCI**	2.0 (2.0-3.0)		907 (37.0)	
**ASA II/III/IV/V**	482/478/8/1 (49.7/49.3/0.8/0.1)		969 (39.6)	
**Adenocarcinoma**	2450 (100.0)		2450 (100.0)	
**Differentiation**				
Well	127		2239 (91.4)	
Moderately	872	
Poor	1240	
**Stage**	**Clinical**	**Pathological**	**Clinical**	**Pathological**
Tis/0	15 (1.3)	104 (4.3)	1183 (48.3)	2431 (99.2)
T1	154 (13.0)	515 (21.2)		
T2	235 (19.9)	391 (16.1)		
T3	703 (61.7)	1330 (54.7)		
T4	49 (4.1)	91 (3.7)		
N0	455 (39.0)	979 (40.1)	1166 (47.6)	2441 (99.6)
N1	657 (56.3)	697 (28.6)		
N2	45 (3.9)	411 (16.8)		
N3	9 (0.8)	354 (14.5)		
**Mean (s.d.) no. of lymph nodes excised**	26.0 (18.2)	1967 (80.3)		
**Mean (s.d.) no. of positive lymph nodes**	3.4 (5.5)	1969 (80.4)		
**V/N invasion**	431 (46.5)	927 (37.8)		
**R1**	256 (20.0)	1279 (52.2)		
**Neoadjuvant therapy**	1061 (44.1)	2368 (96.7)		
**Median (range) follow-up (months)**	26 (0–245)^a^	2450 (100.0)		
**Median survival (months)**	39 (95%CI 36–43)	2450 (100.0)		

Data are *n* (%) unless otherwise stated. CCI, Charlson Comorbidity Index (non-age adjusted); V/N, vascular/neural invasion.

### Statistical analysis

Model validation was dependent on the concordance of test variables between the model and available variables within the validation dataset. Validation was performed according to the published eligibility criteria within each study. Missing data were dealt with by imputing the mode for categorical variables, and the median for continuous variables^13^. Risk stratification scoring systems were assessed using Kaplan–Meier curves and the log-rank test. Individualized prediction scoring models were evaluated by plotting calibration curves of predicted against actual survival. Where possible, model performance was further evaluated using receiver operating characteristic (ROC) curves and the corresponding area under the curve (AUC). Statistical analysis was performed using SPSS statistics version 27.0 (IBM, Armonk, New York, USA), with *P* < 0.05 considered to signify statistical significance.

## Results

Of the 8133 studies identified through the electronic search 17 were eligible for inclusion^14–30^. A PRISMA diagram of study selection is provided in *Fig. S1*. Included studies cumulatively assessed 27 460 patients during the period 1988 to 2019. The characteristics of the included studies are presented in *Table 2*. Further study characteristics and details of variables assessed and utilized within models are provided in *Tables S2 and S3*. The commonest features included in models to predict survival were T stage (11 studies), tumour grade (10 studies), N stage (nine studies), and patient age (eight studies). In five studies, patient cohorts were prospectively sourced solely for the development of the intended prognostic models. The remaining studies used pre-existing data registries, including the Surveillance, Epidemiology, and End Results Program (SEER)^18,21,23,26,28,30^, the National Cancer Database (NCDB)^24^, trial datasets^19^, and internal hospital datasets^22,31^. The inclusion of two studies in which the proposed model was intended to predict lymph node metastasis^16,20^ was based on the concurrent association of this outcome with survival that was demonstrated by the authors.

**Table 2:** Characteristics of included studies

Author	Year	Patient no. (training/validation)	Histology	Tumour site	Treatment	Outcome	Method of classification	References
**Deans *et al.***	2007	220/—	AC/SCC	O/GOJ*	Surgery + any	OS	Nomogram	^14^
**Lagarde *et al.***	2007	364/—	AC	O/GOJ	Surgery	DSS	Nomogram	^15^
**Barbour *et al.***	2010	85/—	AC (T1)	O/GOJ	Surg	LNM, OS, DSS	Grades (I–IV)	^16^
**Langer *et al.***	2014	360/—	AC	O	Surgery + nC†	OS	Grades (A, B, C)	^17^
**Eil *et al.***	2014	824/int‡	AC/SCC	O	Surgery ± nCRT	OS	Risk calculator	^18^
**Shapiro *et al.***	2016	626/int‡	AC/SCC	O/GOJ	Surgery + nCRT	OS	Nomogram	^19^
**Davison *et al.***	2016	210/39	AC (T1)	O/GOJ	Surgery	LNM, OS, TRG	Grades (I–IV)	^20^
**Cao *et al.***	2016	4109/145	AC/SCC	O	Surgery ± nRT	OS	Nomogram	^21^
**Lindenmann *et al.***	2017	174/—	AC/SCC	O	Surgery + nCRT	OS, PFS	Grade (I, II)	^22^
**Zhou *et al.***	2015	953/181	AC	GOJ	Surgery+?	OS	Nomogram	^23^
**Gabriel *et al.***	2017	7179/1795	AC	O	Surgery ± nCRT	OS	Risk calculator	^24^
**Zhang *et al.***	2017	355/—	AC	GOJ	Surgery	OS	Nomogram	^25^
**Xie *et al.***	2018	1948/476	AC/SCC	O/GOJ	Surgery + nRT	DSS	Nomogram	^26^
**Goense *et al.***	2018	373/—	AC	O	Surgery + nCRT	PFS, OS	Nomogram	^27^
**Liu *et al.***	2019	1090/728	AC	GOJ	Surgery + nRT	OS	Nomogram	^28^
**Hagens *et al.***	2020	660/—	AC/SCC	O	Surgery + nCRT	OS	Nomogram	^29^
**Du *et al.***	2020	3198/1368	AC/SCC	O	Surgery ± nCRT	DSS, OS	Nomogram	^30^

* Includes gastric cancer. †Thirty-one patients also received neoadjuvant chemotherapy. ‡Int, internal validation; nC, neoadjuvant chemotherapy; nCRT, neo-adjuvant chemoradiotherapy; nRT, neo-adjuvant radiootherapy; AC, adenocarcinoma; SCC, squamous cell carcinoma; O, oesophageal; GOJ, gastroesophageal junction; OS, overall survival; DSS, disease-specific survival; LNM, lymph node metastasis; TRG, tumour regression grade; PFS, progression-free survival.

### Appraisal of studies

Critical appraisal of included studies was performed in accordance with the CHARMS checklist. Eleven studies adopted either a prospective or retrospective cohort design, while seven studies used data from a national data registry. One study used a retrospective case–control design. A summary of the risk of bias within included studies, as determined by the CHARMS checklist, is provided in *Table S4*. Notably, none of the 17 papers achieved the highest CHARMS standards in terms of sample size, missing data, and model development, and only one paper achieved the best rating in candidate predictors. In contrast, interpretation and discussion were performed well in 15 of 17 studies.

### Assessment of model performance

**Fig. 1:**
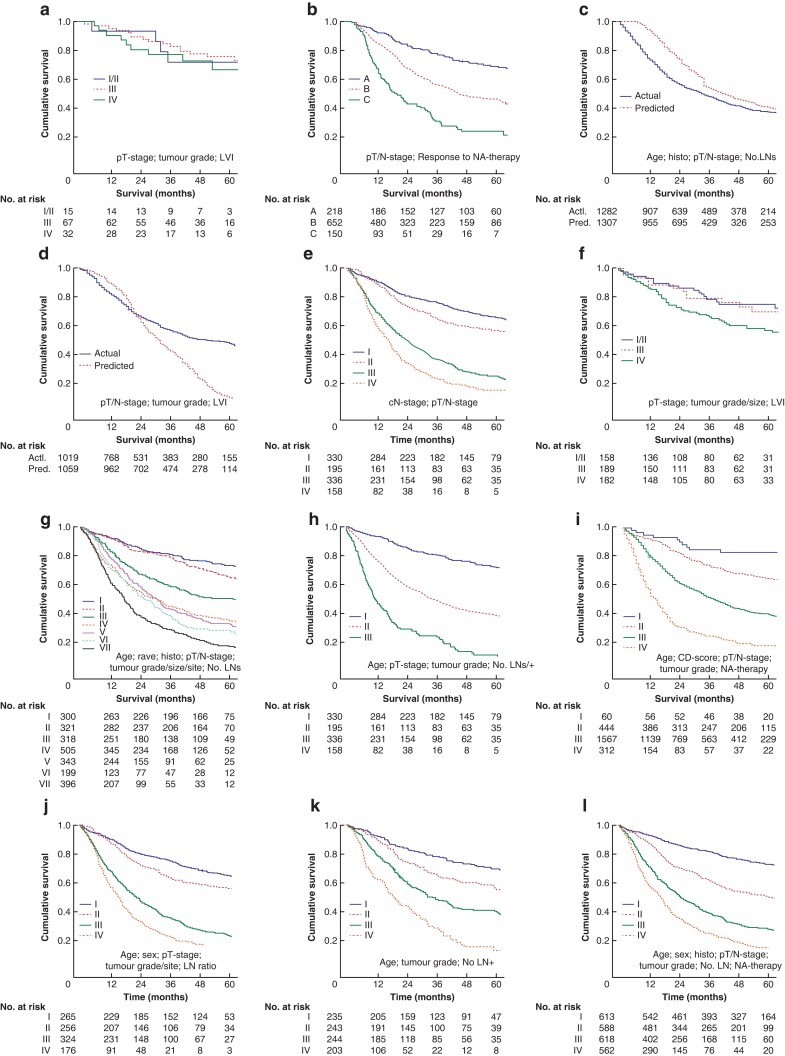
Kaplan–Meier plots of predicted survival **a** Barbour *et al.*, stratification of survival based on nodal metastasis risk grade (grades I/II were considered as a single grade; *P* = 0.859)^16^. **b** Langer *et al.*, stratification of survival based on prognosis score (A = 3, B = 4–5, C = 6; *P* < 0.001)^17^. **c** Eil *et al.*, predicted and actual survival of patients who underwent surgery alone (*P* < 0.001)^18^. **d** Eil *et al.*, predicted and actual survival of patients who underwent neoadjuvant therapy and surgery (*P* < 0.001)^18^. **e** Shapiro *et al.*, stratification of survival based on nomogram score (I = 0–4, II = 5–8, III = 9–11, IV ≥ 12; *P* < 0.001)^19^. **f** Davison *et al.*, stratification of survival based on nodal metastasis risk grade (grades I/II were considered as a single grade; *P* < 0.009)^20^. **g** Cao *et al.*, stratification of survival based on nomogram score (I = 0.0–6.6, II = 6.7–9.0, III = 9.1–11.0, IV = 11.1–13.8, V = 13.9–16.7, VI = 16.8–18.7, VII ≥ 18.8; *P* < 0.001)^21^. **h** Zhou *et al.*, stratification of survival based on nomogram score (I = 0–93, II = 94–187, III = 188–280; *P* < 0.001)^23^. **i** Gabriel *et al.*, stratification of survival based on nomogram score (I = 1, II = 2, III = 3, IV = 4–5; *P* < 0.001)^24^. **j** Xie *et al.*, stratification of survival based on nomogram score (I = 0–88, II = 89–142, III = 143–172, IV ≥ 173; *P* < 0.001)^26^. **k** Liu *et al.*, stratification of survival based on nomogram score (I = 0–48, II = 49–64, III = 65–88, IV ≥ 89; *P* < 0.001)^28^. **l** Du *et al.*, stratification of survival based on nomogram score (I = 0–99, II = 100–158, III = 159–200, IV ≥ 200; *P* < 0.001)^30^. Box contains variables included in the model. pT-stage, pathological T-stage. pN-stage, pathological N-stage. LVI, lymphovascular invasion. NA-therapy, neoadjuvant therapy. Histo, histology. No. LNs, number of lymph nodes examined. No LNs/+, total number of lymph nodes examined and number of positive lymph nodes. CD-score, Charlson-Deyo comorbidity score

**Fig. 2:**
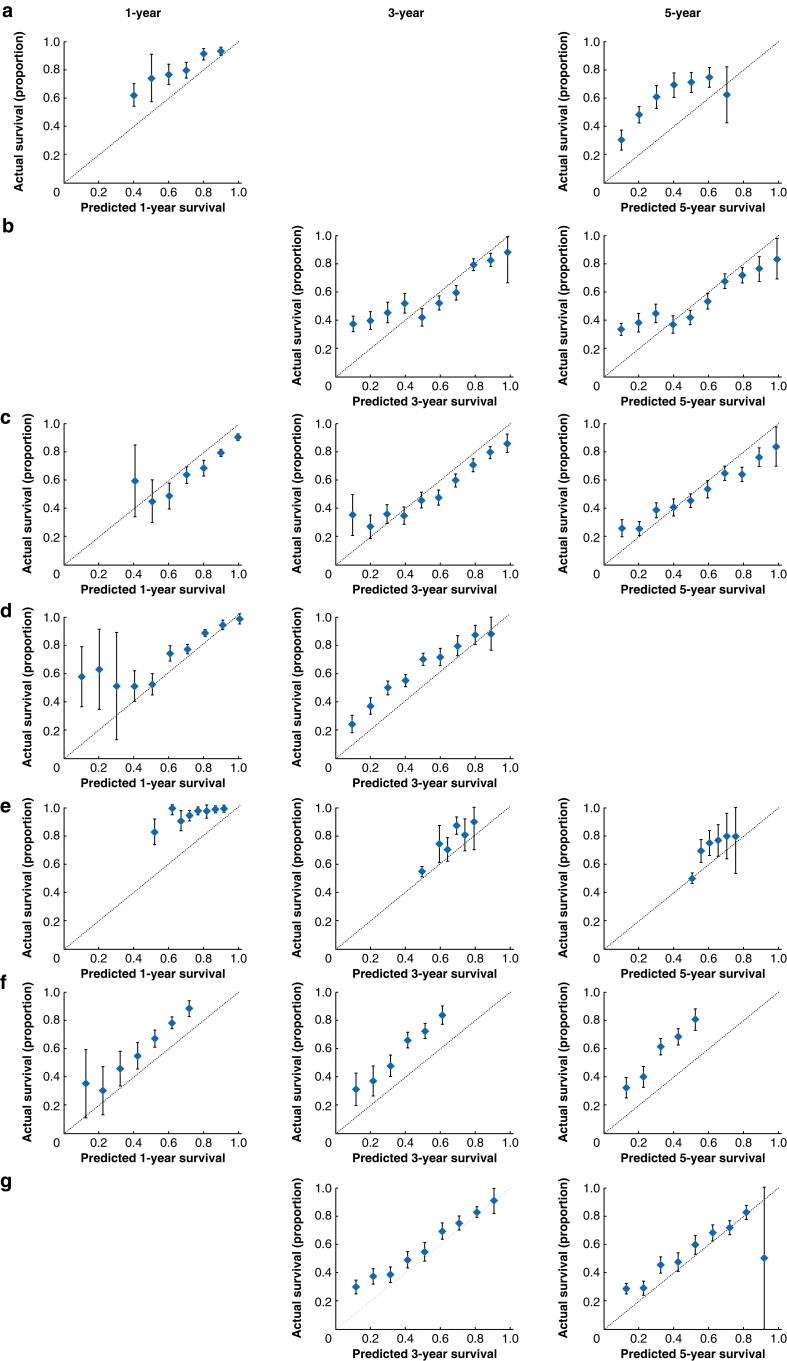
Calibration plots curves for predicted survival **a** Shapiro *et al.*^19^. **b** Cao *et al.*^21^. **c** Zhou *et al.*^23^. **d** Gabriel *et al.*^24^. **e** Xie *et al.*^26^. **f** Liu *et al.*^28^. **g** Du *et al.*^30^

Of the 17 studies identified from the literature search, 11 presented models that were suitable for testing against the independent validation dataset^16–21,23,24,26,28,30^. Details of models that were assessed, including a comparison of survival outcomes with the validation dataset, are presented in *Table 3* and *Figs 1 and 2*, with full details provided in *Table S5*. With the exception of the models reported by Barbour *et al.*^16^ (*Fig. 1a*) and Eil *et al*.^18^ (*Fig. 1c,d*), all models were able to predict survival successfully in patients within the independent validation dataset. The model published by Davison *et al*.^20^ demonstrated poor discrimination of groups I/II and III *versus* group IV (*Fig. 1f*). In terms of individual predictions of survival, this was, generally speaking, more accurate for longer surviving patients, with ROC-AUC ranging from 0.658 to 0.705 (*Table 3*); however, some models tended to under-predict survival^19,24^.

**Table 3:** Comparison of survival outcomes

	Original cohort			Validation cohort					
	No.	OS (%)*	5-year OS for model subgroups (%)	No.	Median survival (months)†	5-year OS (%)	5-year OS for model subgroups (%)	*P*	AUC†
**Barbour *et al.***	85	77	I 97 II 87 III 50 IV 29	114	137 (121–153)	75	I/II 73 — III 78 IV 72	0.859	—
**Langer *et al.***	360	—	A 64 B 42 C 18	1061	48 (40–57)	57	A 73 B 57 C 33	<0.001	—
**Eil *et al.***	824	—	—	2450	39 (36–43)	49	—	—	—
**Shapiro *et al.***	626	—	—	1061	48 (40–57)	57	—	<0.001	0.672 (0.639–0.705) *P* < 0.001
**Davison *et al.***	210	—	I 89 II 87 III 65 IV 42	544	102 (84–120)	69	I/II 77 — II 74 III 63	<0.001	—
**Cao *et al.***	4109	40	I 84 II 69 III 54 IV 40 V 22 VI 11 VII 5	2450	39 (36–43)	49	I 75 II 69 III 54 IV 40 V 42 VI 39 VII 33	<0.001	0.658 (0.637–0.680) *P* < 0.001
**Zhou *et al.***	953	38	I 65 II 37 III 18	2450	39 (36–43)	49	I 76 II 46 III 27	<0.001	0.696 (0.675–0.717) *P* < 0.001
**Gabriel *et al.***	7179	54‡	—	2450	39 (36–43)	56	—	<0.001	0.68 (0.661–0.703) *P* < 0.001
**Xie *et al.***	1948	—	—	1061	48 (40–57)	57	—	<0.001	0.673 (0.640–0.705) *P* < 0.001
**Liu *et al.***	1090	—	—	964	47 (38–56)	57	—	<0.001	0.677 (0.643–0.711) *P* < 0.001
**Du *et al.***	3198	41	—	2450	39 (36–43)	49	—	<0.001	0.705 (0.684–0.726) *P* < 0.001

* Values are for 5-year overall survival (OS) unless otherwise stated. †Values in parentheses are 95% confidence intervals. ‡Value is for survival at 3 years. AUC, area under the receiver operating characteristic curve.

## Discussion

This is the first study to systematically identify and attempt to validate published models for the prediction of survival in oesophageal adenocarcinoma. Owing to the generally poor prognosis of oesophageal cancer^2^, it is vital to have accurate prognostic information so that patients and clinicians can make informed treatment choices. This makes a clear case for the benefit of well-constructed and reliable predictive models. While the TNM system stratifies patients into groups with significantly different outcomes^32^, the majority of patients fall into stage III, limiting its real-world utility. This systematic review identified 17 published prediction models, that assessed a combined 50 variables. These studies were of variable methodological quality, with none reaching the highest standards in several core assessment criteria: sample size, missing data, and model development^10^. Ten of the 17 models were derived from existing cohorts of patients and seven from national registries.

Eleven of the 17 models could be tested using a large multicentre cohort of patients from the UK, USA, Ireland, and the Netherlands, including cohorts from the OCCAMS Consortium^11,12^ and the newly formed POEM Biomarkers Consortium. This mixed cohort represents a good test of their predictive power. It was reassuring that nine of the 11 models successfully validated; however, complete separation of all prognostic groups was not seen in two studies^16,20^. It is also notable that one of the models that failed to validate^16^ had one of the smallest assessment cohorts (85 patients) and was one of the oldest studies (1991 to 2008).

Models that predicted individual survival did reasonably well but with a tendency to under-predict short-term survival. The AUCs for the models clustered around 0.65 to 0.70, which is considered poor-to-fair accuracy. Therefore, this raises the question of how these models could be used in clinical scenarios. It is noteworthy that none of the models presented herein is currently in routine clinical use. Existing models may have limited use on an individual patient basis but could potentially be used to stratify patients into different treatment groups: neoadjuvant chemotherapy and surgery *versus* surgery alone, for example. It is notable that many were designed for a subset of patients with oesophageal adenocarcinoma (e.g. T1 or neoadjuvant chemoradiotherapy) that potentially makes them more restricted in their utility. However, in one such model, Davison *et al.* were able to subdivide the T1 group to some degree, identifying a poorer prognosis group (IV) that may warrant more aggressive treatment^20^.

A further limitation of existing models is, for the most part, their reliance upon knowledge of pathological staging. For patients and clinicians, it would be more informative to have information regarding prognosis at the time of diagnosis, when it may have the largest impact on clinical decision-making and treatment planning. Future work should therefore place a greater emphasis on the identification of pretreatment prognostic markers.

The strengths of this study include the carefully constructed systematic review following published Cochrane Library and MOOSE guidelines^9^. The validation cohort was large, mixed, and multinational; however, as the individual databases differed in terms of the data recorded it was not possible to use all the patients to validate every model. In particular, the small numbers of patients used to test the model published by Barbour *et al*.^16^ may have been linked to its failure to validate; however, the test set was larger than the cohort used to generate the original model. It was not possible to assess the performance of all identified models, owing to a limited number of variables within the validation dataset. A further limitation was the decision to exclude models that included novel biomarkers such as immunohistochemistry and genetic markers^11^. Such models are likely to have an important role to play in the future, but it was decided to concentrate on those models that could be immediately implemented by surgical centres and therefore, by definition, were models that could be tested easily.

This systematic review and validation of models designed to predict survival in oesophageal adenocarcinoma demonstrates that the models already have potential to stratify patients in a more granular way than standard TNM staging. However, none has yet achieved widespread adoption, with this study being the first time any of these models have been tested in other cohorts. To develop a robust and reliable model it is important to avoid as many of the potential sources of bias as possible and to generate and test the model in a large and multicentre cohort. This avoids the danger of overfitting the data to local outcomes^33^. While these 17 models have added to the field, it has not been translated into widespread adoption, and therefore they have failed to alter management decisions or improve outcomes in the real world. This must be the goal for any predictive model. While we did not include models that included biomarkers it is notable that, despite many being proposed^34^, none of these are in widespread use either, very likely for the same reasons that these clinical models have not been adopted.

Future work developing and validating predictive models in oesophageal cancer must concentrate on adopting robust and bias-free methodologies, suitably sized and representative patient cohorts, and, most importantly, externally validate the models in independent patient groups. If the work is carried out in this way, it will increase the likelihood of adoption and therefore improve the chance of improving patients’ outcomes.

## Collaborators

We are very grateful to the POEMS Biomarker and OCCAMS Consortium collaborators that provided anonymised data for this study and reviewed the final manuscript: R.C. Fitzgerald, R.H. Hardwick, J.M.H. Bennett, P.M. Safranek, A.C. Hindmarsh, N. Carroll, and J.R. O’Neill (Cambridge Oesophagogastric Centre/Cambridge University); T. Underwood, R. Walker, and J. Harrington (Southampton University); R. Turkington (Queens University Belfast); K.S. Nason, J.M. Davison, and J.D. Luketich (University of Pittsburgh); J. Reynolds and J. O’Sullivan (Trinity St. James’s Cancer Institute, Dublin); R. Skipworth (Edinburgh Royal Infirmary); S. Darnton and R. Steyn (Birmingham Heartlands Hospital); J. Going and M. McKernan (Glasgow Royal Infirmary); R. Stuart (Ross Hall Hospital, Glasgow); M. Moorghen, J. Blazeby, and C. P. (Barham Bristol Royal Infirmary); J. Shapiro, W.N.M. Dinjens, K. Biermann, and B.P.L. Wijnhoven (Erasmus University Medical Center, Rotterdam); and C. Rajaguru, N. Imrit, and N. Maynard (Oxford Radcliffe Hospitals NHS Trust).

## Supplementary Material

znac044_Supplementary_Data
