# Supplementary material for: Systematic review and validation of clinical models predicting survival after oesophagectomy for adenocarcinoma
Source: Br J Surg. 2022 Mar 2;109(5):418–25. doi: 10.1093/bjs/znac044 (PMC10364693; doi:10.1093/bjs/znac044)
Supplement: znac044_Supplementary_Data [file znac044_supplementary_data.zip › Supplementary_material.docx]

**Supplementary material**

**Table S1.** Electronic search strategy

| (“cancer staging” [Mesh] or “clinical model” or “clinical marker” or “staging system” or “prognostic factor” or “clinical variable” or “tumo?r characteristic” or “demographics” or “nomogram” or “risk stratification”)  AND  (“disease free survival” [Mesh] or “survival” [Mesh] or “cancer survival” [Mesh] or “mortality” or “death” or “prognosis” or “outcome”)  AND  (“?esophag* cancer” or “?esophag* neoplasm” or “?esophag* malignancy” or “?esophag* adenocarcinoma” or “?esophag* carcinoma” or “gastroesophag* cancer” or “gastroesophag* neoplasm” or “gastroesophag* malignancy” or “gastroesophag* adenocarcinoma” or “gastroesophag* carcinoma”). |
| --- |

**Table S2.** Characteristics of included studies

| **Author** | **Year** | **Data origin** | **Study period** | **Design** | **Patient No.**  **(training)** | **Patient No.**  **(validation)** | **Sex (M)** | **Histology** | **Tumour**  **site** | **Treatment** | **Outcome** | **Method of classification** | **Quality of evidence** ^d^ | **Ref** |
| --- | --- | --- | --- | --- | --- | --- | --- | --- | --- | --- | --- | --- | --- | --- |
| **Deans** | 2007 | UK | 2002-2004 | Cohort (P) | 220 | None | 145 | Adeno/SCC | O/GOJ^a^ | Surg+any | OS | Nomogram | 4 | ^14^ |
| **Lagarde** | 2007 | Netherlands | 1993-2003 | Cohort (P) | 364 | None | 309 | Adeno | O/GOJ | Surg | DSS | Nomogram | 4 | ^15^ |
| **Barbour** | 2010 | Australia | 1991-2008 | Cohort (P) | 85 | None | 79 | Adeno | O/GOJ (T1) | Surg | LNM,OS,DSS | I/II, III, IV ^c^ | 4 | ^16^ |
| **Langer** | 2013 | Germany | 1995-2010 | Cohort (P) | 360 | None | 346 | Adeno | O | Surg+nC^b^ | OS | A, B, C | 4 | ^17^ |
| **Eil** | 2014 | USA | 1995-2005 | National registry (R) | 824 | Internal | 328 | Adeno/SCC | O | Surg±nCRT | OS | Risk calculator | 4 | ^18^ |
| **Shapiro** | 2016 | Netherlands | 2001-2013 | Cohort (R) | 626 | Internal | 492 | Adeno/SCC | O/GOJ | Surg+nCRT | OS | Nomogram | 4 | ^19^ |
| **Davison** | 2016 | USA | 1996-2012 | Cohort (R) | 210 | 39 | 179 | Adeno | O/GOJ (T1) | Surg | LNM,OS,TRG | I/II, III, IV ^c^ | 4 | ^20^ |
| **Cao** | 2016 | USA/China | 1988-2007 | National registry (R) | 4109 | 145 | 1497 | Adeno/SCC | O | Surg±nRT | OS | Nomogram | 4 | ^21^ |
| **Lindenmann** | 2016 | Austria | 2003-2011 | Cohort (R) | 174 | None | 148 | Adeno/SCC | O | Surg+nCRT | OS,PFS | I/II | 4 | ^22^ |
| **Zhou** | 2015 | USA/China | 1988-2011 | National registry (R) | 953 | 181 | 912 | Adeno | GOJ | Surg+? | OS | Nomogram | 4 | ^23^ |
| **Gabriel** | 2017 | USA | 2006-2012 | National registry (R) | 7179 | 1795 | 7931 | Adeno | O | Surg±nCRT | OS | Risk calculator | 4 | ^24^ |
| **Zhang** | 2017 | China | 2010-2011 | Cohort (R) | 355 | None | 281 | Adeno | GOJ | Surg | OS | Nomogram | 4 | ^25^ |
| **Xie** | 2018 | USA | 1998-2013 | National registry (R) | 1948 | 476 | 2058 | Adeno/SCC | O/GOJ | Surg+nRT | DSS | Nomogram | 4 | ^26^ |
| **Goense** | 2018 | USA | 2006-2016 | Cohort (P) | 373 | None | 519 | Adeno | O | Surg+nCRT | PFS,OS | Nomogram | 4 | ^27^ |
| **Liu** | 2019 | USA | 1998-2013 | National registry (R) | 1090 | 728 | 1579 | Adeno | GOJ | Surg+nRT | OS | Nomogram | 4 | ^28^ |
| **Hagens** | 2020 | Netherlands | 2004-2019 | Cohort (R) | 660 | None | 528 | Adeno/SCC | O | Surg+nCRT | OS | Nomogram | 4 | ^29^ |
| **Du** | 2020 | USA | 2004-2013 | National registry (R) | 3198 | 1368 | 3881 | Adeno/SCC | O | Surg±nCRT | DSS,OS | Nomogram | 4 | ^30^ |
| ^a^Includes gastric cancer. ^b^31 patients also received neoadjuvant chemotherapy. ^c^Original model describes 4 grades. ^d^ Quality of evidence score (1 [highest] to 5 [lowest]), modified from the Oxford Centre for Evidence-based Medicine for ratings of individual studies. P, prospective. R, retrospective. M, male. Adeno, adenocarcinoma. SCC, squamous cell carcinoma. O, oesophageal. GOJ, Gastroesophageal junction. OS, overall survival. DSS, disease specific survival. PFS, progression free survival. LNM, lymph node metastasis. TRG, tumour regression grade | | | | | | | | | | | | | | |
|  | | | | | | | | | | | | | | |

**Table S3.** Details of variables assessed within prognostic models

|  | **Deans** | **Lagarde** | **Barbour** | **Langer** | **Eil** | **Shapiro** | **Davison** | **Cao** | **Lindenmann** | **Zhou** | **Gabriel** | **Zhang** | **Xie** | **Goense** | **Liu** | **Hagens** | **Du** |
| --- | --- | --- | --- | --- | --- | --- | --- | --- | --- | --- | --- | --- | --- | --- | --- | --- | --- |
| **PATIENT CHARACTERISTICS** | | | | | | | | | | | | | | | | | |
| Age | **♦** | **♦** | **♦** | **-** | **♦** | **♦** | **-** | **♦** | **-** | **♦** | **♦** | **♦** | **♦** | **♦** | **♦** | **♦** | **♦** |
| Sex | **♦** | **♦** | **♦** | **♦** | **♦** | **♦** | **♦** | **♦** | **♦** | **♦** | **♦** | **♦** | **♦** | **♦** | **♦** | **♦** | **♦** |
| Race | **-** | **-** | **-** | **-** | **-** | **-** | **-** | **♦** | **-** | **-** | **-** | **-** | **♦** | **-** | **-** | **-** | **♦** |
| Body weight | **-** | **-** | **-** | **-** | **-** | **-** | **-** | **-** | **♦** | **-** | **-** | **-** | **-** | **-** | **-** | **-** | **-** |
| BMI | **♦** | **-** | **-** | **-** | **-** | **-** | **-** | **-** | **-** | **-** | **-** | **♦** | **-** | **♦** | **-** | **♦** | **-** |
| Weight loss | **♦** | **-** | **-** | **-** | **-** | **♦** | **-** | **-** | **-** | **-** | **-** | **-** | **-** | **♦** | **-** | **-** | **-** |
| Rate of weight loss | **♦** | **-** | **-** | **-** | **-** | **-** | **-** | **-** | **-** | **-** | **-** | **-** | **-** | **-** | **-** | **-** | **-** |
| Level of dietary intake | **♦** | **-** | **-** | **-** | **-** | **-** | **-** | **-** | **-** | **-** | **-** | **-** | **-** | **-** | **-** | **-** | **-** |
| Dysphagia score | **♦** | **-** | **-** | **-** | **-** | **-** | **-** | **-** | **-** | **-** | **-** | **-** | **-** | **-** | **-** | **-** | **-** |
| Karnowski score | **♦** | **-** | **-** | **-** | **-** | **-** | **-** | **-** | **-** | **-** | **-** | **-** | **-** | **-** | **-** | **-** | **-** |
| CD-comorbidity score | **-** | **-** | **-** | **-** | **-** | **-** | **-** | **-** | **-** | **-** | **♦** | **-** | **-** | **-** | **-** | **♦** | **-** |
| Serum CRP | **♦** | **-** | **-** | **-** | **-** | **-** | **-** | **-** | **♦** | **-** | **-** | **-** | **-** | **-** | **-** | **-** | **-** |
| Serum albumin | **-** | **-** | **-** | **-** | **-** | **-** | **-** | **-** | **♦** | **-** | **-** | **♦** | **-** | **-** | **-** | **-** | **-** |
| Prealbumin | **-** | **-** | **-** | **-** | **-** | **-** | **-** | **-** | **-** | **-** | **-** | **♦** | **-** | **-** | **-** | **-** | **-** |
| Haemoglobin | **-** | **-** | **-** | **-** | **-** | **-** | **-** | **-** | **-** | **-** | **-** | **♦** | **-** | **-** | **-** | **-** | **-** |
| NLR | **-** | **-** | **-** | **-** | **-** | **-** | **-** | **-** | **-** | **-** | **-** | **♦** | **-** | **-** | **-** | **-** | **-** |
| PLR | **-** | **-** | **-** | **-** | **-** | **-** | **-** | **-** | **-** | **-** | **-** | **♦** | **-** | **-** | **-** | **-** | **-** |
| PNI | **-** | **-** | **-** | **-** | **-** | **-** | **-** | **-** | **-** | **-** | **-** | **♦** | **-** | **-** | **-** | **-** | **-** |
| **TUMOUR CHARACTERISTICS** | | | | | | | | | | | | | | | | | |
| Tumour histology | **-** | **-** | **-** | **-** | **♦** | **♦** | **-** | **♦** | **♦** | **-** | **-** | **-** | **♦** | **♦** | **♦** | **♦** | **♦** |
| T Stage | **-** | **♦^p^** | **♦^p^** | **♦^p^** | **♦^p^** | **♦^p^** | **♦^p^** | **♦^p^** | **♦^p^** | **♦^p^** | **♦^c^** | **-** | **♦^p^** | **♦^c^** | **♦^p^** | **♦^p^** | **♦^p^** |
| Depth of submucosal invasion | **-** | **-** | **♦^p^** | **-** | **-** | **-** | **-** | **-** | **-** | **-** | **-** | **-** | **-** | **-** | **-** | **-** | **-** |
| Tumour grade | **♦** | **♦** | **♦** | **♦** | **-** | **♦** | **♦** | **♦** | **♦** | **♦** | **♦** | **♦** | **♦** | **♦** | **♦** | **-** | **♦** |
| Tumour size | **-** | **♦** | **♦** | **-** | **-** | **♦** | **♦** | **♦** | **-** | **-** | **-** | **♦** | **♦** | **-** | **-** | **-** | **-** |
| Tumour site | **-** | **-** | **♦** | **-** | **-** | **♦** | **-** | **♦** | **-** | **-** | **-** | **♦** | **♦** | **-** | **-** | **-** | **♦** |
| Tumour necrosis | **-** | **-** | **-** | **-** | **-** | **-** | **-** | **-** | **-** | **-** | **-** | **-** | **-** | **-** | **-** | **-** | **-** |
| Barrett’s mucosa | **-** | **♦** | **♦** | **-** | **-** | **-** | **-** | **-** | **-** | **-** | **-** | **-** | **-** | **-** | **-** | **-** | **-** |
| Sarcoidosis lesion | **-** | **-** | **-** | **-** | **-** | **-** | **-** | **-** | **-** | **-** | **-** | **-** | **-** | **-** | **-** | **-** | **-** |
| N Stage | **-** | **♦^p^** | **-** | **♦^p^** | **♦^p^** | **♦^c/p^** | **-** | **♦^p^** | **♦^p^** | **-** | **♦^c^** | **-** | **-** | **♦^c^** | **-** | **♦^c/p^** | **♦^p^** |
| No. of positive LN | **-** | **♦** | **-** | **-** | **-** | **-** | **-** | **-** | **-** | **♦** | **-** | **-** | **♦** | **-** | **♦** | **-** | **-** |
| No. of LN examined | **-** | **-** | **-** | **-** | **♦** | **-** | **-** | **♦** | **-** | **♦** | **-** | **-** | **♦** | **-** | **-** | **-** | **♦** |
| LN ratio | **-** | **♦** | **-** | **-** | **-** | **-** | **-** | **-** | **-** | **-** | **-** | **-** | **♦** | **-** | **♦** | **-** | **-** |
| LN morphology grade | **-** | **-** | **-** | **-** | **-** | **-** | **-** | **-** | **-** | **-** | **-** | **-** | **-** | **-** | **-** | **-** | **-** |
| Capsular LNI | **-** | **-** | **-** | **-** | **-** | **-** | **-** | **-** | **-** | **-** | **-** | **-** | **-** | **-** | **-** | **-** | **-** |
| Extracapsular LNI | **-** | **♦** | **-** | **-** | **-** | **-** | **-** | **-** | **-** | **-** | **-** | **-** | **-** | **-** | **-** | **-** | **-** |
| Truncal LNI | **-** | **♦** | **-** | **-** | **-** | **-** | **-** | **-** | **-** | **-** | **-** | **-** | **-** | **-** | **-** | **-** | **-** |
| Capsular LN fibrosis | **-** | **-** | **-** | **-** | **-** | **-** | **-** | **-** | **-** | **-** | **-** | **-** | **-** | **-** | **-** | **-** | **-** |
| Central LN fibrosis | **-** | **-** | **-** | **-** | **-** | **-** | **-** | **-** | **-** | **-** | **-** | **-** | **-** | **-** | **-** | **-** | **-** |
| Lymphovascular invasion | **-** | **-** | **♦** | **-** | **-** | **-** | **♦** | **-** | **-** | **-** | **-** | **-** | **-** | **-** | **-** | **-** | **-** |
| Lymphatic invasion | **-** | **-** | **-** | **-** | **-** | **-** | **-** | **-** | **-** | **-** | **-** | **-** | **-** | **-** | **-** | **-** | **-** |
| Vascular transformation | **-** | **-** | **-** | **-** | **-** | **-** | **-** | **-** | **-** | **-** | **-** | **-** | **-** | **-** | **-** | **-** | **-** |
| Multifocal neoplasia | **-** | **-** | **♦** | **-** | **-** | **-** | **-** | **-** | **-** | **-** | **-** | **-** | **-** | **-** | **-** | **-** | **-** |
| Lymphangiosis carcinomatosis | **-** | **-** | **-** | **-** | **-** | **-** | **-** | **-** | **-** | **-** | **-** | **-** | **-** | **-** | **-** | **-** | **-** |
| M Stage | **-** | **-** | **-** | **♦** | **-** | **-** | **-** | **-** | **-** | **-** | **-** | **-** | **-** | **-** | **-** | **-** | **-** |
| TNM stage | **♦^c^** | **-** | **-** | **-** | **-** | **-** | **-** | **-** | **-** | **-** | **-** | **♦^p^** | **-** | **-** | **-** | **-** | **-** |
| Resection margin | **-** | **♦** | **-** | **♦** | **-** | **♦** | **-** | **-** | **-** | **-** | **-** | **-** | **-** | **-** | **-** | **-** | **-** |
| **TREATMENT CHARACTERISTICS** | | | | | | | | | | | | | | | | | |
| Surgical approach | **-** | **-** | **-** | **-** | **-** | **♦** | **-** | **-** | **-** | **-** | **-** | **-** | **-** | **-** | **-** | **♦** | **-** |
| ASA classification | **-** | **-** | **-** | **♦** | **-** | **-** | **-** | **-** | **-** | **-** | **-** | **-** | **-** | **-** | **-** | **♦** | **-** |
| NA-therapy | **-** | **-** | **-** | **♦** | **-** | **-** | **-** | **♦** | **♦** | **-** | **♦** | **-** | **♦** | **♦** | **-** | **-** | **♦** |
| Tumour response to NA-therapy | **-** | **-** | **-** | **♦** | **-** | **♦** | **-** | **-** | **-** | **-** | **-** | **-** | **-** | **♦** | **-** | **-** | **-** |
| Adjuvant therapy | **-** | **-** | **-** | **-** | **-** | **-** | **-** | **-** | **♦** | **-** | **-** | **-** | **-** | **-** | **-** | **-** | **-** |
| **♦** = assessed and included in model; **♦**= assessed but not included in model; - = not assessed or included in model; ♦^c^= clinical staging; ♦^p^= pathological staging. BMI, body mass index. CD, Charlson-Deyo comorbidity score. CRP, C-reactive protein. NLR, neutrophil-lymphocyte ratio. PLR, platelet-lymphocyte ratio. PNI, prognostic nutritional index. TNM, tumour / node / metastasis. LN, lymph nodes. LNI, lymph node invasion. ASA, American Society of Anaesthesiologists score. NA, neoadjuvant. | | | | | | | | | | | | | | | | | |

**Table S4.** CHARMS Checklists

| **Domain** | **Deans** | **Lagarde** | **Barbour** | **Langer** | **Eil** | **Zhou** | **Davison** | **Cao** | **Lindernmann** | **Gabriel** | **Zhang** | **Shapiro** | **Du** | **Goense** | **Hagens** | **Liu** | **Xie** |
| --- | --- | --- | --- | --- | --- | --- | --- | --- | --- | --- | --- | --- | --- | --- | --- | --- | --- |
| **SOURCE OF DATA** | Low | Low | Low | Moderate | High | High | Moderate | High | Moderate | High | Moderate | Low | High | Low | Moderate | High | High |
| **PARTICIPANTS** | Moderate | Moderate | Low | Moderate | Low | Moderate | Moderate | Low | Moderate | Low | Low | Low | Moderate | Low | Low | Low | Moderate |
| **OUTCOME(S) TO BE PREDICTED** | Low | Moderate | High | Low | Moderate | Low | High | Low | Low | Low | Low | Low | Low | Low | Low | Moderate | Low |
| **CANDIDATE PREDICTORS**  **(OR INDEX TESTS)** | Moderate | Moderate | Moderate | Moderate | Moderate | Moderate | Moderate | Moderate | Moderate | Low | Moderate | Moderate | Low | Moderate | Moderate | Low | Low |
| **SAMPLE SIZE** | Moderate | Moderate | High | High | High | Moderate | Moderate | Moderate | Moderate | Moderate | Moderate | Moderate | Moderate | Moderate | Moderate | Moderate | Moderate |
| **MISSING DATA** | High | Moderate | Moderate | High | Moderate | High | Moderate | Moderate | Moderate | Moderate | Moderate | Low | Moderate | Low | Low | Moderate | High |
| **MODEL DEVELOPMENT** | Moderate | High | High | High | Moderate | Moderate | Moderate | Moderate | Moderate | Moderate | Moderate | Moderate | Moderate | Low | Low | Moderate | Moderate |
| **MODEL PERFORMANCE** | Moderate | Low | High | Moderate | Moderate | Low | Moderate | Low | High | Low | Low | Moderate | Moderate | Moderate | Moderate | Moderate | Moderate |
| **MODEL EVALUATION** | Moderate | Low | Moderate | High | Moderate | Low | Low | Low | High | Low | High | High | Low | Low | Low | Low | Low |
| **RESULTS** | Low | Low | Moderate | Low | Low | Low | Moderate | Low | High | Low | Low | Low | Low | Low | Low | Low | Low |
| **INTERPRETATION AND DISCUSSION** | Moderate | Low | Low | Low | Low | Low | Low | Low | Low | Low | Moderate | Low | Low | Low | Low | Low | Low |

|  | **Low** |
| --- | --- |
|  |  |
|  | **Moderate** |
|  |  |
|  | **High** |

**
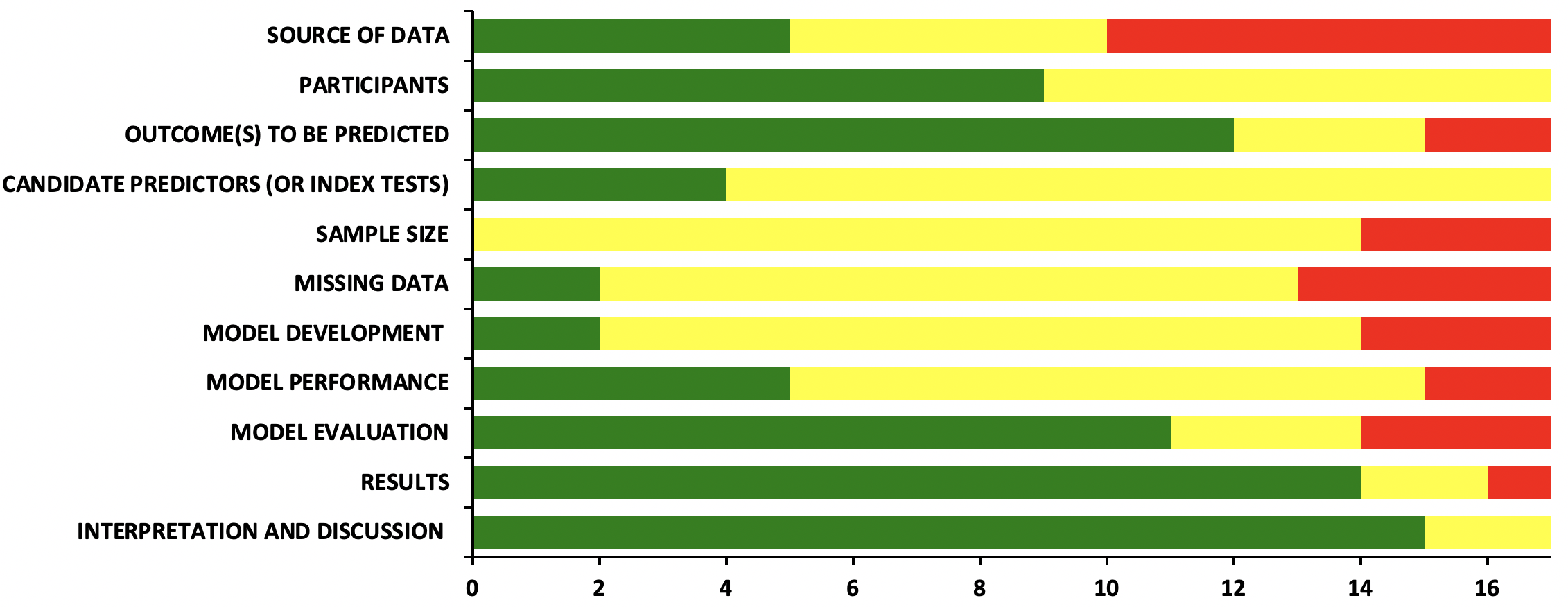
**

**Table S5**. Comparison of survival outcomes

| **Author** | **Original cohort** | | | | |  | **Validation cohort** | | | | | | |
| --- | --- | --- | --- | --- | --- | --- | --- | --- | --- | --- | --- | --- | --- |
|  | **Patient No.** | **Follow-up (months)**^a^ | **Median survival**  **(months)** | **OS**^c^ | **5yr OS**  **for model subgroups** |  | **Patient**  **No.** | **Follow-up (months)**^a^ | **Median survival**  **(months)** | **5yr OS** | **5yr OS**  **for model subgroups** | **P** | **AUC** |
| **Barbour** | 85 | 59 | - | 77% | I 97%; II 87%; III 50%; IV 29% |  | 114 | 51 (1-189) | 137 (95%CI 121-153) ^b^ | 75% | I/II 73%; III 78%; IV 72% | 0·859 | - |
| **Langer** | 360 | - | 37 (95%CI 28-45) | - | A 64%; B 42%; C 18% |  | 1061 | 27 (0-194) | 48 (95%CI 40-57) | 57% | A 73%; B 57%; C 33% | <0.001 | - |
| **Eil** | 824 | - | 24 | - | - |  | 2450 | 26 (0-245) | 39 (95%CI 36-43) | 49% | - | - | - |
| **Shapiro** | 626 | - | - | - | - |  | 1061 | 27 (0-194) | 48 (95%CI 40-57) | 57% | - | <0·001 | 0·672 (95% CI 0·639 to 0·705)  P<0·001 |
| **Davison** | 210 | - | - | - | I 89; II 87%; III 65%; IV 42% |  | 544 | 43 (0-193) | 102 (95%CI 84-120) | 69% | I/II 77%; II 74%; III 63% | <0·001 | - |
| **Cao** | 4109 | 28 (3-276) | - | 40% | I 84%; II 69%; III 54%; IV 40%;  V 22%; VI 11%; VII 5% |  | 2450 | 26 (0-245) | 39 (95%CI 36-43) | 49% | I 75%; II 69%; III 54%; IV 40%;  V 42%; VI 39%; VII 33% | <0·001 | 0·658 (95% CI 0·637 to 0·680)  P<0·001 |
| **Zhou** | 953 | 25.5^b^ | - | 38% | I 65%; II 37%; III 18% |  | 2450 | 26 (0-245) | 39 (95%CI 36-43) | 49% | I 76%; II 46%; III 27% | <0·001 | 0·696 (95% CI 0·675 to 0·717)  P<0·001 |
| **Gabriel** | 7179 | 49 (0-105) | 42.6 | 54%^d^ | - |  | 2450 | 26 (0-245) | 39 (95%CI 36-43) | 56% | - | <0·001 | 0·682 (95% CI 0·661 to 0·703)  P<0·001 |
| **Xie** | 1948 | 33.7 | - | - | - |  | 1061 | 27 (0-194) | 48 (95%CI 40-57) | 57% | - | <0·001 | 0·673 (95% CI 0·640 to 0·705)  P<0·001 |
| **Liu** | 1090 | - | - | - | - |  | 964 | 27 (0-187) | 47 (95%CI 38-56) | 57% | - | <0.001 | 0·677 (95% CI 0·643 to 0·711)  P<0·001 |
| **Du** | 3198 | 78 (95%CI 75.9-80.1) | - | 40.5% | - |  | 2450 | 26 (0-245) | 39 (95%CI 36-43) | 49% | - | <0·001 | 0·705 (95% CI 0·684 to 0·726)  P<0·001 |
| 95%CI, 95% confidence interval. OS, overall survival. AUC, area under the receiver operating characteristic curve. ^a^Values are median (range) unless otherwise stated. ^b^Average. ^c^Values are for five-year OS unless otherwise stated. ^d^Value is for 3 year survival. | | | | | | | | | | | | | |

**Figure S1.** PRISMA flow chart of literature search.

**Records excluded after review of title and abstracts**

**n = 8091**

**Additional full text records included after hand searching of reference lists**

**n = 1**

**Studies excluded after assessment of full text**

**n = 26**

**Records screened**

**n = 8133**

**Records identified through electronic database searching (search performed on 11/07/2018)**

**OVID (EMBASE, MEDLINE):**

**Total = 8133**

**Full text articles assessed for eligibility**

**n = 42**

**Full text articles included after electronic search**

**n = 16**

**Studies included**

**n = 17**
